# Midlife alcohol consumption and longitudinal brain atrophy: the PREVENT-Dementia study

**DOI:** 10.1007/s00415-020-10000-8

**Published:** 2020-06-20

**Authors:** Michael J. Firbank, John T. O’Brien, Karen Ritchie, Katie Wells, Guy Williams, Li Su, Craig W. Ritchie

**Affiliations:** 1 Institute of Neuroscience, Newcastle University, Campus for Ageing and Vitality, Nuns Moor Road, Newcastle upon Tyne, NE4 5PL UK; 2 Department of Psychiatry, University of Cambridge, Level E4 Cambridge Biomedical Campus, Box 189, Cambridge, CB2 0SP UK; 3 INSERM, Montpellier, France; 4 Centre for Dementia Prevention, University of Edinburgh, Edinburgh, UK; 5 The Centre for Psychiatry, Imperial College London, 7th floor Commonwealth Building, Du Cane Road, London, W12 0NN UK; 6 Department of Clinical Neurosciences and Wolfson Brain Imaging Centre, University of Cambridge, Cambridge Biomedical Campus, Cambridge, CB2 0SP UK

**Keywords:** MRI, Alcohol, Mid-life, Brain volume, Longitudinal, Atrophy

## Abstract

**Background and aims:**

Consensus is lacking on whether light to moderate consumption of alcohol compared to abstinence is neuroprotective. In this study, we investigated the relationship between self-reported alcohol use and brain volume change over 2 years in middle-aged subjects.

**Methods:**

A sample of 162 subjects (aged 40–59 at baseline) from the PREVENT-Dementia programme underwent MRI scans on two separate occasions (mean interval 734 days; SD 42 days). We measured longitudinal rates of brain atrophy using the FSL Siena toolbox, and change in hippocampal volume from segmentation in SPM.

**Results:**

Controlling for age and sex, there were no significant associations of either total brain, ventricular, or hippocampal volume change with alcohol consumption. Adjusting for lifestyle, demographic and vascular risk factors did not alter this.

**Conclusions:**

We did not find any evidence of influence of alcohol consumption on changes in brain volume over a 2-year period in 40–60-year-olds.

## Introduction

High consumption of alcohol has consistently been linked with dementia and brain degeneration; however, several, but not all, studies have suggested that moderate consumption vs. abstinence is a protective factor against dementia [1, 2]. Some studies of the association of alcohol consumption with brain volume have similarly found protective effects of light to moderate drinking [3], whilst others have found that brain volume tends to linearly decrease with increased alcohol intake [4]. Topiwala et al. [5] found higher alcohol consumption associated with smaller hippocampi, as contrasted with Downer et al. [6] who found larger hippocampi for those with moderate consumption. A systematic review concluded that hippocampal volume was reduced in those with problematic alcohol use [7]. All these studies used cross-sectional imaging data, and are thus not sensitive to any ongoing neurodegeneration.

The aim of this study was to investigate alcohol consumption and longitudinal changes in brain volume in middle-aged adults. We investigated whole brain and ventricular volume as well as hippocampal volume, due to its association with both alcohol dependence and dementia. We hypothesised that increased alcohol consumption would be associated with brain atrophy.

## Materials and methods

### Subjects

The protocol has been described in detail elsewhere [8]. A total of 193 participants aged 40–59, of whom 168 had repeat MRI were recruited through multiple sources. Initially they were identified from the dementia register database held at a London National Health Service (NHS) Trust, part of the UK National Health Service. Other participants were recruited via the Join Dementia Research website (https://www.joindementiaresearch.nihr.ac.uk/), through information about the study on the Internet and public presentations. The study aimed to recruit approximately half of the subjects with a parental family history of dementia and half without. All MRI scans obtained on participants were formally reported by an experienced consultant neuroradiologist. Any subject with significant incidental findings (e.g., tumour, stroke) was excluded. Approval for the study was given by the NHS Research Ethics Committee London Camberwell St-Giles (REC reference: 12/LO/1023). All participants provided informed written consent.

### Imaging

Participants underwent multimodal 3 T structural magnetic resonance imaging on a single scanner (Siemens Verio) including volumetric T1-weighted scans (176 slices, 1.0 × 1.0 mm, 1.0 mm slice thickness, repetition time = 2300 ms, echo time = 2.98 ms, flip angle 9°). Scans were repeated after approximately 2 years on the same scanner using the same protocol. Imaging findings from these participants have been previously reported [9–11].

Percentage brain and ventricular volume change between the two scans were determined using the FSL SIENA program (https://fsl.fmrib.ox.ac.uk/fsl/fslwiki). Values were then divided by the time between scans to give rates per year. SPM12 (https://www.fil.ion.ucl.ac.uk/spm) was used to segment the brain into grey matter, white matter and CSF, and spatially normalise the images to standard space. To determine hippocampal volume, the hippocampal region of interest (ROI) from the AAL template [12] was used to determine the mean of the normalised grey matter segmentation image. Left and right were averaged, and the longitudinal change was determined by subtracting the baseline measurement from repeat. Total intracranial volume (ICV) was calculated from the sum of grey matter, white matter and CSF segmentation at baseline. All output was manually checked to ensure correct brain segmentation, and alignment of baseline/repeat scans.

### Lifestyle variables

Demographic data and information on drinking and smoking status were collected from case report forms which hold participants’ data collected at interview. We also included data from the participants’ medical history, which included history of alcoholism and diabetes. Participants were classified as current smokers or non-smokers (never and former).

We used the Lifetime of Experiences Questionnaire (LEQ) [13] to extract data about physical, social and cultural activity (art, music, hobbies) from the age of 30 years to present. The three physical questions used from the LEQ asked participants how often they engaged in activities that were (1) mildly energetic (such as walking), (2) moderately energetic and (3) vigorous (such as running). For each activity, participants were asked to indicate their response on the six-point Likert scale from Never to Daily. We dichotomised the scores as to whether people did the activities more vs. less frequently than once a month.

Alcohol consumption in units per week was determined using the Scottish Collaborative Group Food Frequency Questionnaire version 7.1 (https://www.foodfrequency.org) completed by the participant at baseline and repeat visit. The questionnaire asks for the typical amount and frequency of consumption of 175 food and drink items over the last 2–3 months. We took the average of baseline and repeat alcohol consumption, except for eight participants for whom repeat values were missing, in which case we used the baseline value. Following previous research [5] and the UK guidelines of 14 units per week as a safe limit (https://www.drinkaware.co.uk/alcohol-facts/alcoholic-drinks-units/latest-uk-alcohol-unit-guidance/), alcohol usage was divided into abstaining (< 1 unit/week), light (> = 1 and < 7 units/week), moderate (> = 7 and < 14 units/week), high (> = 14 and < 21 units/week) and very high (> = 21 units per week). No distinction was applied between men and women in this classification. For a subgroup analysis on male and female separately, we divided alcohol use into three groups: (0–5), (> 5–15), (> 15) units per week to avoid having too few male subjects in each group.

The Framingham risk score was used to calculate 10-year cardiovascular risk from current smoking status, systolic BP, BP medication status, sex, age at baseline, total and HDL cholesterol and diagnosis of diabetes. Social class was determined using the UK National Statistics Socio-economic classification (NS-SEC).

### Statistical analysis

Brain volume data and clinical variables were analysed with R version 3.5.2. Independent *t* tests were used to compare groups for continuous variables. Linear regression was used to investigate association of alcohol with brain atrophy rates. Total brain and ventricular volume change were expressed as percentage of baseline volume (as calculated by SIENA). For hippocampal analysis, we included ICV as a covariate to adjust for head size. Residuals were checked by eye to verify normality, heteroscedasticity and absence of nonlinear associations.

## Results

Of the 193 participants with T1 MR scans at baseline, 168 had a repeat scan. One participant was excluded as an outlier (ventricular change was negative, 3SD below mean). We excluded one subject due to missing alcohol consumption data, and four with a history of alcohol dependence, leaving 162 in the main analysis.

Mean age at baseline was 52 year (SD 5.4), and mean interval between scans 734 (SD 42) days. There were 24 alcohol abstainers, 42 light, 44 moderate, 20 high and 32 very high consumers of alcohol. The highest intake group included six persons (four male) drinking > 50 units/week. There were 112 female and 50 male participants, with men having higher alcohol consumption than women 19.7 (SD 21.3) vs. 10.9 (SD 12.1) units/week *t*_63.5_ = 2.74, *p* = 0.008, Welch two-sample *t* test.

Mean Framingham risk score was 6.6% (SD 5.5%, min = 0.9%, max = 47%). There were 25/162 participants who engaged in artistic, musical activities or hobbies at least once a fortnight; 149/161 who met family or friends; 151/162 engaged in mild, 128/162 moderate, and 76/162 vigorous activities. 85 participants had one or both parents with dementia, and there were six current smokers. Mean years of education was 16.1 (SD 3.4). There were 36 people in social class one, 56 in class two, 22 in class three, 10 in class four, 7 in classes five–seven and 26 in class eight, with 5 people not classified. 35 people had a medical history of depression, two of whom had a recurrence during the study, and a further two had incident depression. Supplementary Tables 1 and 2 present these data split by alcohol consumption. The moderate consumption group had the lowest Framingham score, which was significantly lower than the abstainer group (*p* = 0.02, Tukey HSD post hoc). The abstainer group had significantly fewer years of education than the moderate (*p* = 0.007 Tukey HSD post hoc) and the very high group (*p* = 0.03 Tukey HSD post hoc).

**Fig. 1:**
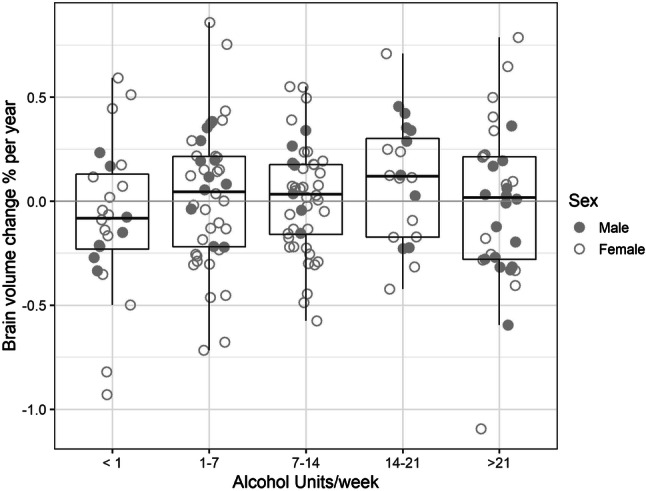
Percentage brain volume change per year for different alcohol intake groups. Negative values indicate brain shrinkage

In linear models including alcohol consumption, age, sex (and intracranial volume for hippocampus), there was no significant effect of alcohol on brain change (Table 1), although there was a trend (*p* = 0.09) of association between drinking 14–21 units of alcohol and preserved total brain volume (Table 1, Fig. 1). Addition of other factors (education, midlife activities, social class, vascular risk factors, family history of dementia) did not alter this lack of significant association between alcohol and longitudinal brain change [supplementary Tables 3, 4]. The addition of the four subjects with a history of alcohol dependence did not qualitatively alter the results. As sex was a strong predictor of atrophy, we also repeated the linear model analysis in male and female participants separately. In this, we split alcohol usage into three groups due to relatively low numbers of male participants. The demographics and results of the linear model are in Supplementary Tables 5–7. There were no significant associations of brain volume change with alcohol in this analysis.

**Table 1:** Linear model predictors of annual percentage ventricle change, total brain volume change and hippocampal volume change

	Percent ventricle change per year F_6,155_ = 6.4; *p* < 0.001		Percent brain volume change per year F_6,155_ = 2.98; *p* < 0.009		Hippocampal volume change per year F_7,154_ = 3.58; *p* = 0.001	
	Beta (SE)		Beta (SE)		Beta (SE)	
Age	0.16 (0.04)	*t* = 4.59; *p* = < 0.001**	− 0.017 (0.005)	*t* = − 3.68; *p* = < 0.001**	− 0.11 (0.60)	*t* = − 0.19; *p* = 0.848
Male sex	1.60 (0.43)	*t* = 3.75; *p* = < 0.001**	0.042 (0.056)	*t* = 0.75; *p* = 0.452	0.70 (9.77)	*t* = 0.07; *p* = 0.943
1–7 units/week	0.51 (0.62)	*t* = 0.82; *p* = 0.414	0.073 (0.081)	*t* = 0.90; *p* = 0.368	10.68 (10.33)	*t* = 1.03; *p* = 0.303
7–14 units/week	0.72 (0.62)	*t* = 1.16; *p* = 0.247	0.055 (0.081)	*t* = 0.69; *p* = 0.493	0.09 (10.48)	*t* = 0.01; *p* = 0.993
14–21 units/week	-0.49 (0.73)	*t* = − 0.67; *p* = 0.502	0.162 (0.095)	*t* = 1.70; *p* = 0.091	2.73 (12.37)	*t* = 0.22; *p* = 0.826
> 21 units/week	0.10 (0.66)	*t* = 0.15; *p* = 0.883	0.061 (0.085)	*t* = 0.71; *p* = 0.478	9.52 (10.96)	*t* = 0.87; *p* = 0.386
Intracranial volume					− 118.5 (35.0)	*t* = − 3.39; *p* = < 0.001**

Alcohol values are referenced to the abstainer group (< 1 unit alcohol per week)

**p < 0.001

## Discussion

Contrary to our hypothesis, we did not observe any significant association between alcohol consumption and longitudinal brain volume changes. Rather, we saw a non-significant trend of 14–21 units of alcohol (vs. abstinence) associated with preserved total brain volume.

Studies of alcohol on cognition or brain structure have confounds due to social and demographic factors, with age, years of education, and social class all being linked to both alcohol consumption and brain volume. Our study, looking at brain volume change over 2 years within individuals overcomes to some extent these confounds, and including the lifestyle, demographic and vascular health factors in our analysis did not change our findings.

Combined with the previous conflicting reports on the benefit or otherwise of mild to moderate alcohol consumption, our data suggest at least that consumption of 7–21 units per week is not associated with marked brain atrophy over a 2-year period in midlife.

Limitations of the study include that alcohol consumption was estimated from subject report, and the relatively short follow-up of 2 years. The participants were mostly female, limiting the extrapolation to the general population.

In summary, we did not find any evidence of influence of alcohol consumption on changes in brain volume over a 2-year period in 40–60-year-olds.
